# Effects of Hay, Baleage, and Soybean Hulls Waste Used as Supplemental Feeds on the Nutritional Profile of Grass-Finished Beef

**DOI:** 10.3390/foods11233856

**Published:** 2022-11-29

**Authors:** Lucas Krusinski, Isabella C. F. Maciel, Selin Sergin, Vijayashree Jambunathan, Esha Garg, Andrea J. Garmyn, Sukhdeep Singh, Chad A. Bitler, Jason E. Rowntree, Jenifer I. Fenton

**Affiliations:** 1 Department of Food Science and Human Nutrition, Michigan State University, 469 Wilson Rd, East Lansing, MI 48824, USA; 2 Department of Animal Science, Michigan State University, 474 S Shaw Ln, East Lansing, MI 48824, USA; 3 Department of Plant, Soil, and Microbial Sciences, Michigan State University, 1066 Bogue St, East Lansing, MI 48824, USA; 4 Greenacres Foundation Inc., Cincinnati, OH 45242, USA

**Keywords:** cattle feed, conserved forages, fatty acids, grassland, meat quality, minerals, pasture, sustainability

## Abstract

Grass-finished beef (GFB) has demonstrated wide nutritional variations with some GFB having a considerably higher *n*-6:*n*-3 ratio compared to grain-finished beef. To better understand these variations, the current study investigated the effects of commonly used supplemental feeds on the nutritional profile of GFB. This two-year study involved 117 steers randomly allocated to one of four diets: (1) grass+hay (G-HAY), (2) grass+baleage (G-BLG), (3) grass+soybean hulls (G-SH), and (4) baleage+soybean hulls in feedlot (BLG-SH). Feed samples were analyzed for their nutritional value, and beef samples underwent analysis for fatty acids (FAs), vitamin E, minerals, lipid oxidation, and shear force. FAs were measured by GC-MS, vitamin E was analyzed chromatographically, minerals were analyzed by ICP-MS, and lipid oxidation was measured via a thiobarbituric acid reactive substances (TBARS) assay. G-SH beef had the highest *n*-6:*n*-3 ratio (*p* < 0.001), while BLG-SH beef contained less vitamin E (*p* < 0.001) and higher TBARS values (*p* < 0.001) compared to the other groups. G-HAY beef contained more long-chain *n*-3 polyunsaturated FAs compared to the other groups (*p* < 0.001). In conclusion, G-HAY beef had the most beneficial nutritional profile, while soybean hulls increased the *n*-6:*n*-3 ratio of beef.

## 1. Introduction

The market for grass-finished beef (GFB) is growing with retail sales of pasture-raised beef increasing from $17 million in 2012 to $272 million in 2016 [1,2]. The consumer reasoning for these growing sales is complex. However, sustainable food production is driving food choices for savvy consumers [3,4]. GFB appeals to consumers who are interested in a healthier product for human consumption and the environmental considerations [5]. Leading organizations and platforms such as the EAT Lancet commission recommend to drastically reduce red meat consumption for health and environmental reasons [6]; however, the type of production system is generally ignored in this recommendation [2]. GFB remains an underexplored alternative to attain sustainability goals [7]. For one, GFB is more consistent with health recommendations. Compared to conventional grain-finished beef, GFB contains more omega-3 (*n*-3) fatty acids (FAs) including eicosapentaenoic acid (EPA) and docosahexaenoic acid (DHA), twice as much conjugated linoleic acid (CLA), and 25% more polyunsaturated FAs (PUFAs) [3,8,9,10,11]. GFB also contains less omega-6 (*n*-6) PUFAs, less total fat, and less cholesterol-raising saturated FAs (SFAs) [12,13]. GFB has an *n*-6:*n*-3 ratio of 1.5:1 compared to 7.7:1 for conventional grain-finished beef [14,15]. Foods containing an *n*-6:*n*-3 ratio closer to 1:1 are recommended by human nutrition health professionals [16,17]. Additionally, consumption of foods containing higher concentrations of phytochemicals, typically fruits and vegetables, is also a key recommendation. In fact, GFB has important antioxidant properties that also contribute to its healthfulness [18,19]. GFB contains three times more vitamin E and 1.5–10 times more β-carotene than grain-finished beef [15,20,21,22]. Some studies suggest that grass-finishing enhances the phenolic content of beef, especially when cattle are grazing on phytochemically biodiverse pastures [2,19,23]. Properly managed complex biodiverse pastures also have the merit to sequester more carbon and to enhance fresh-water systems [24].

The nutritional composition of beef is highly dependent on the feeding system, yet these differences in nutritional quality are generally not reflected on food labels [2,19]. GFB typically means that cattle were fattened solely on grass and forages before slaughter [25]. According to the American Grassfed Association standards, all cattle must be pasture based meaning that grass and forage must be consumed throughout the lifetime of the animal except for milk consumed before weaning. Hay, baleage, and silages may be consumed by the animal when fresh grass and forages are not available due to inclement weather for instance [26]. Supplemental feeds might be needed in some regions where fresh forages are not available year-round. Season, soil composition, weather, and light exposure all play crucial roles in feed quality and availability [19,27,28,29].

Interestingly, a nutritional survey of commercially available GFB published by our group highlighted important differences among beef from grass-finishing systems [21]. The *n*-6:*n*-3 ratio varied from 1.8:1 to as high as 28.3:1 and some GFB was devoid of β-carotene. These differences were hypothesized to be due to a wide variety of feeding practices that were reflected in the nutritional profile of beef [21]. Feeding fresh forages to cattle usually results in the most beneficial nutritional profile of beef [19,21]. However, producers may rely on conserved forages and other supplemental feeds when fresh grass is not available [30]. Unfortunately, conserved forages made by drying (hay) or fermentation (baleage) often display lower nutritional quality compared to fresh forages with lower concentrations of antioxidants and phenolic compounds [31,32]. The processing of fresh forages into conserved feeds results in oxidation of PUFAs and an increase in palmitic acid [33,34]. These changes in the nutritional profile of feeds modify FA metabolism in the rumen, resulting in variations in the FA content of beef [35,36]. Although not allowed by the American Grassfed Association [26], soybean hulls are also used as supplemental feed by some producers in the U.S. [19,21]. The effects of feeding soybean hulls to cattle remain controversial in the literature. Some studies found no differences in CLA, *trans* vaccenic acid (TVA), *n*-3 PUFAs, and the *n*-6:*n*-3 ratio among cattle fed soybean hulls or fresh forage in the finishing phase [20,21]. On the other hand, another study reported that cattle supplemented with soybean hulls had more total fat, less *n*-3 PUFAs, and a higher *n*-6:*n*-3 ratio compared to cattle fed only fescue [37].

Krusinski et al. [19] highlighted that the nutritional profile of GFB is highly variable and depends on a multitude of factors ranging from supplemental feeds to seasonal variations. The present study builds on the work of Bronkema et al. [21] in an attempt to explain the large variations reported among GFB, especially regarding the *n*-6:*n*-3 ratio. With growing interest in assessing the nutritional impact and sustainability of food systems, determining the accurate nutritional value of foods is crucial. The objective of this study was to compare the FA and micronutrient content of GFB fed a diverse pasture mixture and commonly used supplemental feeds to better understand the effects of different feeds on the nutritional profile of GFB.

## 2. Materials and Methods

The animal protocol was reviewed and approved by the Michigan State University Institutional Animal Care and Use Committee (IACUC #202000054).

### 2.1. Experimental Design, Animals, and Diets

This two-year study (2020 and 2021) took place at the Michigan State University Kellogg Biological Station (KBS) located in Hickory Corners, MI (latitude: 42°24′38″ N, longitude: 85°22′45″ W, elevation: 282 m). Sixty steers for each year were randomly allocated to one of four diets: grass supplemented with hay (G-HAY), grass supplemented with baleage (G-BLG), grass supplemented with soybean hulls (G-SH), or baleage and soybean hulls in feedlot (BLG-SH). Three groups for each diet were formed (*n* = 5 animals/replicate; 3 replicates/diet; 15 animals/diet) for each year. Animals were randomly stratified and allocated to one of the three groups in each diet.

In April of each year, 60 Simmental-Angus influenced feeder cattle weighing on average 387 kg (±47 kg) were purchased from the same producer and shipped from Oklahoma to KBS. Upon their arrival at KBS, initial weights were collected, and steers were randomly stratified and assigned to the diets. Steers allocated to the three diets containing grass were kept on pasture and had ad libitum access to a diverse pasture mixture (GRASS) and 4.5 kg of supplemental feed (dry matter; DM) per day per head. Steers kept in the feedlot group had ad libitum access to baleage (BLG) and 4.5 kg of soybean hulls (SH) per day per head. GRASS was a five-species mix of alfalfa (*Medicago sativa*), red clover (*Trifolium pratense* L.), white clover (*Trifolium repens* L.), orchard grass (*Dactylis glomerata* L.), and endophyte-free tall fescue (*Festuca arundinacea*). Dry hay (HAY) was composed of alfalfa (*Medicago sativa*), orchard grass (*Dactylis glomerata* L.), and tall fescue (*Festuca arundinacea*). BLG was a mixture of alfalfa (*Medicago sativa*) and orchard grass (*Dactylis glomerata* L.). Each subgroup for each grass-containing diet was allocated a fenced paddock. In total, each diet was allocated three paddocks, each containing five animals. Each paddock was further divided into sub-paddocks to give time to the pasture to rest and regrow. Animals were rotated three times per week within their paddocks to fresh parcels of grass. The 15 steers in the BLG-SH diet were treated as feedlot cattle and were separated into three pens each containing five animals. One animal died during the first year of the experiment, and two carcasses were misplaced by the slaughterhouse during the second year of the study, bringing the total number of animals for the entire study to 117 (*n* = 117).

### 2.2. Sample Collection and Preparation

#### 2.2.1. Feed Samples

Samples from grazing areas and supplemental feeds were collected every two weeks. The sample collection started in July and ended in late October of each year. No sample collection occurred between April and July 2020 because of COVID-19 restrictions. To stay consistent, the sample collection period was kept the same for 2021. GRASS samples were gathered every two weeks in each sub-paddock immediately before animals had access to the pasture (*n* = 63 for each year, *n* = 126 in total). GRASS samples were collected by randomly cutting three 0.25 m^2^ quadrats to a 5 cm stubble using hand grass clipper scissors. HAY, BLG, and SH were sampled monthly before being distributed to the steers (*n* = 4 of each supplemental feed for each year, *n* = 8 of each in total). Supplemental feeds were sampled monthly instead of bi-weekly because less variations over time in the nutritional profile of these feeds were expected. For proximate analysis, wet weights were recorded, and samples were dried in a forced-air oven (72 h, 55 °C) and ground through a 1 mm screen in a Wiley mill (Arthur H. Thomas, Philadelphia, PA, USA). For FA and phytochemical analysis, a 30 g sub-sample was packed in a Whirl-Pak bag and frozen at −20 °C immediately after collection. In order to obtain representative samples, bag contents were mixed, and 10 g of each replicate was taken before being combined. Feed samples were stored at −20 °C for the length of the trial before being stored at −80 °C once they were brought back to the laboratory. Before further analysis, samples were freeze-dried in a freeze dryer (Harvest Right, North Salt Lake, UT, USA) for 18.5 h, and ground in a Wiley mill (1 mm screen) (Arthur H. Thomas, Philadelphia, PA, USA) with dry ice.

Weather conditions were reported according to Krusinski et al. [38] using the Michigan State University Enviroweather platform at KBS. 30-year normal temperature and precipitation (1991–2020) were reported according to the National Centers for Environmental Information: National Oceanic and Atmospheric Administration website (Gull Lake, MI meteorological station).

#### 2.2.2. Meat Samples

In November of each year, before going to slaughter, steers were weighed again to obtain total weight gain and average daily gain (ADG). Steers were slaughtered in a USDA facility at 18–20 months of age. Body performance and carcass traits (ribeye area, 12th rib back fat, USDA yield grade, and marbling score) were collected by trained personnel 48 h after slaughter. Simultaneously, meat samples (approximately 7.5 to 10 cm in length) were collected from the left-side longissimus lumborum (between 13th rib and first two lumbar vertebra). For FA analysis and thiobarbituric acid reactive substances (TBARS), one steak per carcass was cut into 1 × 1 cm cubes before being flash frozen with liquid nitrogen, put into Whirl-Pak bags, and stored at −80 °C until analysis. For Warner-Bratzler shear force (WBSF), another 2.54 cm-thick steak was cut, vacuum packed, and stored at 4 °C until 14 days postmortem. At 14 days postmortem, the steaks were frozen at −20 °C until WBSF analysis was performed.

### 2.3. Feed Chemical Analysis

#### 2.3.1. Proximate Analysis

The protocol for the feed proximate analysis was previously described by Maciel et al. [39]. Samples were dried at 105 °C in a forced-air oven for 8 h. To determine the ash content, feed samples were oxidized at 500 °C for 6 h in a muffle furnace. Neutral detergent fiber (NDF) was determined according to Mertens [40] with the addition of amylase and sodium sulfite. The protocol described in AOAC [41] was used to determine acid detergent fiber (ADF). Crude protein (CP) was measured according to Hach et al. [42] and gross energy was measured by bomb calorimeter.

#### 2.3.2. Phytochemical Analysis

Chlorophyll A and B were determined as described previously [43]. Briefly, 2 g of lyophilized and ground feed was added to 70% aqueous acetone. The mixture was shaken for 30 min and centrifuged for 20 min (4 °C, 2500 RPM). The upper layer was transferred to a new tube, and the extraction was repeated twice. Compounds were measured using a UV-Vis Double Beam Spectrophotometer (VWR, Radnor, PA, USA) in cuvettes. Readings were recorded at 663 and 646 nm and were used in the following equations:

$${\mathrm{Chlorophyll}\;A\;(C}_{a})=12{.21A}_{663\;}-\;2{.81A}_{646}\;{\mathrm{Chlorophyll}\;B\;(C}_{b})=20{.13A}_{646\;}-\;5{.03A}_{663}$$

To extract phenolic compounds, a modified protocol from Nimalaratne et al. [44] was performed. First, 2 g of freeze-dried and ground feed was added to 20 mL of methanol:distilled water:acetic acid (70:28:2, *v*/*v*/*v*). The mixture was shaken for 30 min and centrifuged for 20 min (4 °C, 2500 RPM). The upper layer was recovered and transferred to a new tube. An additional 20 mL of acetone:distilled water:acetic acid (70:28:2, *v*/*v*/*v*) was added to the original tube before being shaken for 10 min and centrifuged for 15 min (4 °C, 2500 RPM). Both supernatants were combined and stored at 4 °C. The Folin-Ciocalteu assay adapted from Singleton and Rossi [45] was used to measure total phenolic content. A standard curve was made using a 1 mg/mL gallic acid stock solution in methanol. A serial dilution was performed by a factor of two to obtain concentrations ranging from 1 mg/mL to 0.002 mg/mL. Next, 100 μL of Folin-Ciocalteu reagent and 800 μL of 5% sodium bicarbonate were added to the standard curve and to 100 μL of supernatant. The standard curve and the samples were heated at 40 °C for 30 min. Samples were allowed to cool down to room temperature before being plated in triplicates in a 96-well plate. Samples were scanned at 765 nm and compared against the gallic acid standard curve. Values were reported as mg of gallic acid equivalents (GAE)/g of feed.

### 2.4. Fatty Acid Analysis of Feed and Meat

All chemicals were purchased from Sigma-Aldrich (St. Louis, MO, USA) unless otherwise noted.

The FA analysis for feed and meat samples was conducted according to Sergin et al. [46]. A microwave-assisted extraction protocol was performed to extract FAs as reported by Bronkema et al. [21] using a CEM Mars 6 microwave (CEM Corp., Matthews, NC, USA). For this step, 400 mg of ground feed sample or minced meat was added to a microwave vessel containing 8 mL of 4:1 (*v*/*v*) ethyl acetate:methanol solution with 0.1% BHT. The samples underwent extraction with the following settings: 55 °C for 15 min with initial ramp of 2 min at 400 W. Samples were then filtered in another tube containing 3.5 mL of HPLC water before being centrifuged (6 min, 2500 RPM). The upper layer was removed and dried under nitrogen. To resuspend the oil, a 4:1 (*v*/*v*) dichloromethane:methanol solution with 0.1% BHT was used to bring the concentration of each sample to 20 mg of oil/mL.

For the creation of FA methyl esters (FAMEs), a modified version of the protocol by Jenkins [47] was applied. Briefly, 2 mg of oil (100 μL) was resuspended in toluene with 20 μg of internal standard (methyl 12-tridecenoate, U-35M, Nu-Chek Prep, Elysian, MN, USA). Then, 2 mL of 0.5 N anhydrous potassium methoxide was added and the samples were heated at 50 °C for 10 min. Next, 3 mL of methanolic HCl (5%) was added to the samples before being heated at 80 °C for 10 min. Once cool, 2 mL of HPLC water and 2 mL of hexane were added, and samples were centrifuged for 5 min at 2500 RPM. The top layer was moved to another tube and dried under nitrogen to obtain FAMEs. FAMEs were then resuspended in 1 mL of isooctane to get a concentration of 2mg/mL. Samples were transferred to gas chromatography-mass spectrometry (GC-MS) vials with glass inserts.

The PerkinElmer (Waltham, MA, USA) 680/600S GC-MS in electron impact mode (70 eV) equipped with an Agilent Technologies (Santa Clara, CA, USA) HP-88 column (100 m, 0.25 mm ID, 0.2 μM film thickness) was used for the quantification of FAMEs. For feed samples, one μL of sample was injected with the GC temperature set at 250 °C. For meat samples, one μL was injected twice (20:1 split) at two different GC temperatures (175 °C and 150 °C). The temperature settings for both feed and meat samples were as follows: initial temperature at 80 °C for 4 min; ramp 13 °C/min to 175 °C; hold 27 min; ramp 4 °C/min to 215 °C; hold 35 min, and then an initial temperature at 80 °C for 4 min; ramp 13 °C/min to 150 °C; hold 47 min; ramp 4 °C/min to 215 °C; hold 35 min. For meat samples, a third injection followed in splitless mode (0.75 min splitless hold time, 40 mL/min flow exiting the vent). This GC-MS method was adapted from Kramer et al. [48]. Helium was the carrier gas (flow rate of 1 mL/min). MS data were recorded in full scan mode (mass range of m/z 70-400 amu) and the MS transfer line and ion source temperature were set at 180 °C.

MassLynx V4.1 SCN 714 (Water Corporation, Milford, MA, USA) was used for the identification of FAMEs. FAs were identified by retention time and EI mass fragmentation compared to the reference standard containing the Supelco 37 Component FAME Mix with mead acid, docosatetraenoic acid, *n*-3 DPA, *n*-6 DPA, and palmitelaidic acid purchased from Cayman Chemical (Ann Arbor, MI, USA). CLA isomers were identified using the CLA reference standard UC-59M (Nu-Chek Prep, Elysian, MN, USA). FAs not included in the reference standard were identified by elution order and confirmed by the EI mass fragmentation [48]. FAs were quantified using a standard curve including the reference and internal standards. Each FAME concentration was calculated by using the internal standard peak area and analyte peak area compared to the standard curve. C18:1 4*t* and C18:1 5*t* were below the limit of detection, and C18:2 9*c*,12*t* and C18:2 9*t*,12*c* were not separated from the C18:2 11*t*,15*c* peak. Eicosatetraenoic acid (C20:4 *n*-3) was not included in our reference standard and was therefore not reported. FAs were reported in mg/100 g of beef in this manuscript and in percent of total FAs in the Supplementary Tables S1 and S2.

### 2.5. Vitamin E and Mineral Analysis

Protocols by Rettenmaier and Schüep [49] were followed for vitamin E analysis. In brief, 1 g of beef was homogenized in 5 mL of water before being frozen. For extraction, samples were thawed, and a measured aliquot was pipetted out. To precipitate the protein, ethanol was added, and vitamins were extracted with hexane. After being centrifuged, part of the hexane layer was removed and dried under reduced pressure in a vortexing chamber (10 min, 35 °C, 300 mBar vacuum). What remained after evaporation was solubilized in the chromatographic mobile phase and placed in vials. A calibration curve (six points) was made as follows: a vitamin E solution (Sigma-Aldrich, St. Louis, MO, USA) diluted with ethanol (containing BHT) underwent serial dilutions (from 50 μg/mL to 0.2 μg/mL). For the chromatography analysis, a Waters Acquity system and Water Empower Pro Chromatography Manager software (Water Corporation, Milford, MA, USA) were used. An isocratic elution was performed using a mobile phase of acetonitrile:methylene chloride:methanol (70:20:10, *v*/*v*/*v*) and a Symmetry C18, 1.7 μm, 2.1 × 50 mm analytical column (Waters Corporation, Milford, MA, USA). The flow rate was 0.5mL/min and the detection was performed by UV absorption at 292 nm.

Mineral analysis was performed as previously described [50,51]. Briefly, beef samples underwent drying and digestion in an oven (95 °C, overnight) using 10 times the dry tissue mass of nitric acid. A dilution with water to 100 times the dried tissue mass followed. An Agilent 7900 Inductivity Coupled Plasma–Mass Spectrometer (ICP-MS) (Agilent Technologies Inc., Santa Clara, CA, USA) was used for the analysis. A six-point calibration curve was used. Standards of bovine liver and mussels (National Institute of Standards and Technology, Gaithersburg, MD, USA) were used as controls.

### 2.6. Thiobarbituric Acid Reactive Substances (TBARS)

The TBARS assay for food and beverages (Oxford Biomedical Research, Oxford, MI, USA) adapted for a 96-well plate reader was used. First, an eight-point standard curve was created by serial dilution ranging from 0 (only HPLC water) to 3 mg/L malondialdehyde (MDA) (MDA stock solution provided in the kit). Then, 500 mg of minced beef sample was added to 5 mL of HPLC water. Samples were homogenized to obtain a smooth solution. In a microcentrifuge tube, 250 μL of sample solution and 250 μL of the indicator solution (thiobarbituric acid (TBA) and acid solution) were mixed. The indicator solution was also added to the standard curve, and the samples and the curve were set aside for 60 min for the reaction to occur. Samples were then centrifuged at 11,000 RPM for 5 min at room temperature. The aqueous layer was removed and plated in duplicates on a 96-well plate, next to the standard curve. Absorbance was read at 532 nm on a Bio-Tek Synergy HT spectrophotometer (Bio-Tek Instruments, Inc., Winooski, VT, USA). The standard curve was plotted and the MDA concentration for the samples (mg MDA/L) was calculated according to the manufacturer’s instructions.

### 2.7. Warner-Bratzler Shear Force (WBSF)

The protocol for WBSF was previously reported [39]. Briefly, the steaks were cooked to an internal temperature of 71 °C using a preheated clamshell electric grill (George Foreman, Beachwood, OH, USA). The steaks were then cooled down overnight at 4 °C. Six to eight 1.27 cm diameter cores were cut from each steak by paying close attention to cut parallel to the muscle and fibers using a drill mounted corer. Shear force was measured using the TA-XT Texture Analyzer (Stable Micro System Ltd., Godalming, UK) with a V-shaped Warner-Bratzler blade. The blade was moving down at a speed of 20cm/min and cut the sample across the muscle fiber. The purpose of the shear force testing was to measure how much force is required to cut through cooked meat. This should be a representative measure of the ease or difficulty a consumer would have chewing a cooked steak. Most consumer prefer steaks cooked between medium rare and medium well, which is why an internal temperature of 71 °C was chosen for the analysis. The mean of the cores for each sample were used for the statistical analysis.

### 2.8. Statistical Analysis

SAS version 9.4 (SAS Institute Inc., Cary, NC, USA) was used to perform the statistical analysis. Mixed model analysis was performed to test the effect of diet on response variables. In the model, the fixed effect was diet, and the random effects were year and pen nested within year x diet. Each pen was the experimental unit. Post hoc comparison was performed using Tukey’s adjustment, and results were considered significant at *p* < 0.05. Outliers were removed for chlorophyll A and chlorophyll B after running an outlier test. The data satisfied model’s normality and equal variance assumptions. Data are shown as mean ± standard error across mean (SEM).

## 3. Results

### 3.1. Weather Conditions

**Figure 1:**
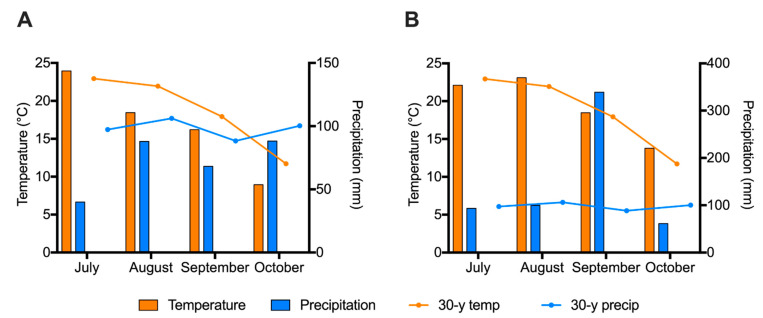
Monthly weather conditions at the experimental site. (**A**) 2020 and (**B**) 2021. 30-y temp: 30-year normal temperature at the experimental site; 30-y precip: 30-year normal precipitation at the experimental site.

Weather conditions at the experimental site for the length of the study are shown in Figure 1. The hottest month in 2020 was July with an average of 23.96 °C. The average temperature in August, September, and October 2020 were all below the 30-year normal. Every month in 2020 was below the 30-year normal for rainfall. In 2021, August was the hottest month with an average temperature of 23.10 °C. July, September, and October 2021 were above the 30-year normal temperature. September 2021 showed unusually high rainfall with 338.87 mm compared to the 30-year normal precipitation of 88.39 mm.

### 3.2. Feeds

#### 3.2.1. Proximate Composition of Feeds

The proximate composition of the feeds is displayed in Table 1. No significant differences were observed between feed types regarding dry matter (DM) (*p* = 0.159). GRASS and BLG had the highest values for ash (*p* < 0.001) and CP (*p* < 0.001), while SH had the lowest values for ash (*p* < 0.001) and HAY had the lowest values for CP (*p* < 0.001). Regarding NDF, SH was higher than GRASS, HAY, and BLG (*p* = 0.004). For ADF, SH was highest while GRASS was lowest (*p* < 0.001). Finally, SH had the lowest amount of energy compared to the other three feed types (*p* < 0.001).

#### 3.2.2. Fatty Acid Composition of the Feeds

The FA profile of the feeds is reported in Table 2. Palmitic acid (C16:0) made up most of the SFA content of the feeds. HAY contained the highest concentration of C16:0, while GRASS and SH contained the lowest (*p* < 0.001). Regarding stearic acid (C18:0), SH and HAY contained the most and GRASS contained the least (*p* < 0.001). The total SFA content was significantly higher in HAY and was lower in GRASS and SH (*p* < 0.001). Total MUFA content was significantly higher in SH and lower in GRASS. BLG and HAY values were in between and were significantly different than SH and GRASS (*p* < 0.001). Regarding PUFAs, the linoleic acid (LA) content was the highest in SH and was lower in GRASS and HAY (*p* < 0.001), while the α-linolenic acid (ALA) content followed the opposite trend with GRASS containing the most and SH containing the least (*p* < 0.001). GRASS contained the highest concentration of total *n*-3 PUFAs and the lowest concentration of *n*-6 PUFAs. SH contained the most *n*-6 PUFAs and the least *n*-3 PUFAs (*p* < 0.001). This resulted in SH having the highest *n*-6:*n*-3 ratio and GRASS having the lowest (*p* < 0.001).

#### 3.2.3. Phytochemical Content of Feeds

**Figure 2:**
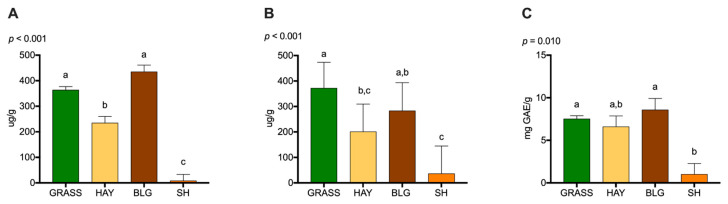
Phytochemical content of feeds. Chlorophyll A (**A**), chlorophyll B (**B**), and total phenols (**C**) found in feed samples. Values reported at means ± standard error. Different letters denote statistical significance at *p* < 0.05 (mixed model analysis, post hoc comparison performed using Tukey’s adjustment, *n* = 126 for GRASS, *n* = 8 for each of the other feeds). GRASS: fresh pasture; HAY: dry hay; BLG: baleage; SH; soybean hulls; GAE: gallic acid equivalent.

The chlorophyll A, chlorophyll B, and total phenols content of the feeds are shown in Figure 2. BLG and GRASS contained the highest levels of chlorophyll A. SH contained the least (*p* < 0.001). GRASS contained more chlorophyll B than HAY and SH, and BLG contained more chlorophyll B than SH (*p* < 0.001). Finally, BLG and GRASS contained more phenols than SH (*p* = 0.010).

### 3.3. Animal Performance and Carcass Traits

Performance and carcass traits are shown in Table 3. Initial weight did not differ between diet groups, which was the goal when assigning animals to each group. Final weight, total gain, and average daily gain (ADG) were all higher in the BLG-SH and G-SH groups, while they were lower in the G-BLG and G-HAY groups (*p* < 0.001). A similar trend was seen regarding hot carcass weight (HCW) with higher weights observed in the G-SH and BLG-SH groups and lower weights observed in the G-HAY and G-BLG groups (*p* = 0.003). Backfat significantly differed by diet (*p* = 0.011). BLG-SH and G-SH had more backfat compared to beef from G-BLG but did not differ from G-HAY. G-HAY had the smallest ribeye area compared to the other three groups (*p* = 0.012). Regarding USDA yield grade, G-BLG had a lower numerical yield grade than G-SH but did not differ from the remaining groups (*p* = 0.032). G-BLG had a lower marbling score than G-SH and BLG-SH but was similar to G-HAY (*p* = 0.004).

### 3.4. Beef Fatty Acids

#### 3.4.1. Saturated and Monounsaturated Fatty Acids

The saturated and monounsaturated FA content of beef is presented in Table 4. No significant differences were observed by diet for total SFAs (*p* = 0.400). Individual SFAs ranging from C10:0 to C20:0 did not differ between groups (*p* > 0.05), but C22:0 was higher in G-HAY compared to the other three groups (*p* < 0.001). No significant differences between groups were observed for total branched-chain FA (BCFA) content or for individual BCFAs (*p* > 0.05). The same trend was observed for total MUFA content and individual *cis*-MUFAs (*p* > 0.05). Regarding *trans*-MUFAs, the only significant difference seen was for C16:1 9*t* which was lower in the BLG-SH group compared to the other three groups (*p* = 0.003). No differences were observed for the total FA content between groups (*p* > 0.05).

#### 3.4.2. Polyunsaturated Fatty Acids and Biohydrogenation Intermediates

**Figure 3:**
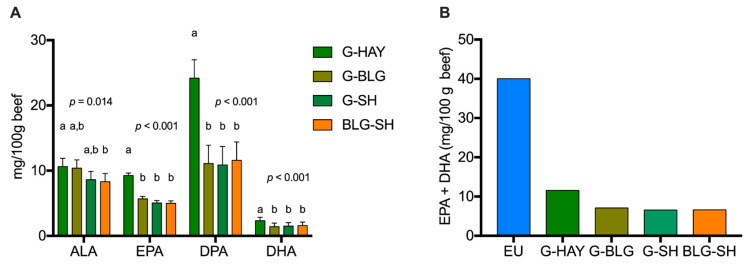
Long-chain *n*-3 PUFAs in beef by diet. (**A**) α-linolenic acid (ALA), eicosapentaenoic acid (EPA), docosapentaenoic acid (DPA), and docosahexaenoic acid (DHA) content of beef by diet. (**B**) Sum of EPA+DHA in beef by diet compared to the European Union (EU) standard to consider a food “a source of *n*-3 PUFAs” [52]. Data shown as means ± standard error. Different letters denote statistical significance at *p* < 0.05 (mixed model analysis, post hoc comparison performed using Tukey’s adjustment, *n* = 117). G-HAY: grass and hay diet; G-BLG: grass and baleage diet; G-SH: grass and soybean hulls diet; BLG-SH: baleage and soybean hulls diet; EU: European Union standard for a food to be considered “a source of *n*-3 fatty acids”.

The PUFA, CLA, and atypical dienes (AD) content of beef are displayed in Table 5. The total PUFA content of beef was higher in the G-HAY group compared to the other three groups (*p* < 0.001). The same was true for the total *n*-3 PUFA content (*p* < 0.001). More specifically, the ALA content of beef was highest in G-HAY and lowest in BLG-SH (*p* = 0.014). All long-chain *n*-3 PUFAs including EPA, DPA, and DHA were higher in beef from the G-HAY group compared to the other three groups (*p* < 0.001). The sum of EPA+DHA was also higher in beef from G-HAY (11.59 mg/100 g of beef) compared to the other three groups, but all four groups had lower amounts of EPA+DHA compared to European Union standards to consider a food “a source of *n*-3 FAs” [52] (Figure 3). No significant differences between groups were observed for *n*-6 PUFAs (*p* > 0.05) except for C22:4 *n*-6 which was higher in beef from G-HAY compared to the other groups (*p* < 0.001). Significant differences in the *n*-6:*n*-3 ratio were also seen; the lowest ratio was seen in the G-HAY group while the highest ratio was seen in beef from G-SH (*p* < 0.001). No differences were observed between the groups for ADs and conjugated linolenic acid (CLnA) (*p* > 0.05). Significant differences were reported for the individual CLA C18:2 9*c*,11*t*/9*c*,7*t* where beef from G-HAY contained the most and beef from BLG-SH contained the least (*p* = 0.015).

### 3.5. Vitamin E and Minerals in Beef

The vitamin E and minerals in beef are presented in Table 6. Vitamin E was significantly lower in beef from the BLG-SH group compared to the other three diets (*p* < 0.001). Selenium, iron, copper, and zinc did not differ between diets (*p* > 0.05). Manganese was higher in beef from G-HAY and G-BLG, and lower in beef from BLG-SH (*p* = 0.002).

### 3.6. Thiobarbituric Acid Reactive Substances and Warner-Bratzler Shear Force Values for Beef

**Figure 4:**
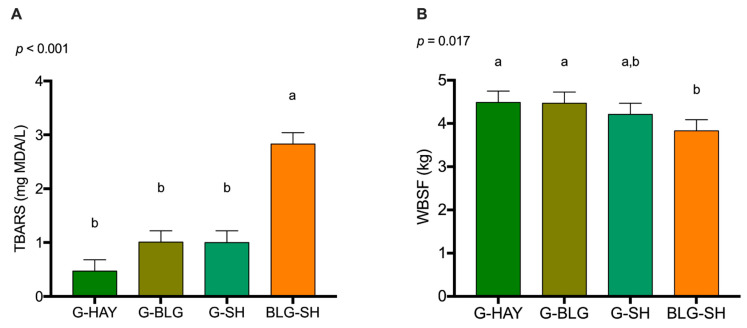
(**A**) Thiobarbituric acid reactive substances (TBARS) and (**B**) Warner-Bratzler Shear Force (WBSF) values of beef by diet. Data shown as means ± standard error. Different letters denote statistical significance at *p* < 0.05 (mixed model analysis, post hoc comparison performed using Tukey’s adjustment, *n* = 117). G-HAY: grass and hay diet; G-BLG: grass and baleage diet; G-SH: grass and soybean hulls diet; BLG-SH: baleage and soybean hulls diet.

The TBARS and WBSF values for beef by diet are displayed in Figure 4. Beef from the BLG-SH group showed higher TBARS values compared to the other three groups (*p* < 0.001). Regarding WBSF values, beef from the BLG-SH group displayed lower values compared to beef from G-HAY and G-BLG (*p* = 0.017).

## 4. Discussion

### 4.1. Feeds

#### 4.1.1. Proximate Composition of the Feeds

The proximate analysis of the feeds indicates that SH are higher in fiber than GRASS, HAY, and BLG. This finding is supported by the literature [53,54]. SH are low in lignin and have a high digestibility potential for ruminants [54]. Therefore, SH provide energy without the management problems associated with high grain diets [53]. In the present study, SH provided less energy than the other three feed types. However, SH were not consumed by the animals in isolation but as a combination with either GRASS or BLG. Poore et al. [54] noted that energy levels of SH were variable in various studies, but because of an associative effect on forage digestion, SH appear to have an effective energy value. GRASS contained the most CP and gross energy compared to the other feeds. These findings confirm previously published results by Krusinski et al. [38] showing that pastures contain more CP and gross energy than conserved forages.

#### 4.1.2. Fatty Acid Profile of the Feeds

Numerous differences in the FA content of the feeds were observed in the current study. GRASS contained more *n*-3 PUFAs in the form of ALA than all the other feeds. This was expected since grasses contain higher concentrations of ALA (50-75% of total FAs) [19,21,38,55]. High levels of PUFAs are found in chloroplasts of green plants (i.e., grasses), which may explain the higher concentrations of ALA [56]. Levels of *n*-3 PUFAs drastically decreased in conserved forages (HAY and BLG). While they still contained more PUFAs than SH, conserved forages usually have reduced nutritional quality compared to fresh grasses. This is due to the drying process of HAY and the fermenting process of BLG which result in the oxidation of PUFAs, especially ALA. More specifically, PUFAs are released from the plant membranes and are then oxidized with exposure to air by lipoxygenases [33]. This process is generally followed by an increase in levels of palmitic acid (C16:0) since SFAs are less prone to oxidation [33,34]. This was confirmed in the present study with HAY and BLG containing more palmitic acid than GRASS. We also reported a higher *n*-6:*n*-3 ratio in SH compared to the other feeds. Bronkema et al. [21] indicated that SH have a higher LA content, thus increasing the *n*-6:*n*-3 ratio. The results presented in the current study confirm these statements since we found higher levels of *n*-6 PUFAs and lower levels of *n*-3 PUFAs in SH compared to the other feeds, thus increasing the *n*-6:*n*-3 ratio. Interestingly, SH contained more MUFA than the other types of feeds (mainly as oleic acid). O’Callaghan et al. [57] showed that adding SH to a concentrate diet decreased levels of oleic acid. Ensiled forages such as BLG have advantages compared to HAY. Ensiling does not greatly impact the FA profile [33,36]. Ensiling forages protects FAs from oxidation, explaining why oxidation in HAY is generally more prevalent [58]. Our results confirm the more beneficial FA profile of BLG compared to HAY; BLG contained more *n*-3 PUFAs and had a lower *n*-6:*n*-3 ratio compared to HAY. However, it is important to note that the feed composition plays a major role in the nutritional profile of feeds. Different plant species have different effects on the FA profile of feeds [32]. A limitation of the current study is the lack of information about the proportion of plant species present in the feeds. Krusinski et al. [38] showed that individual plant species affect the FA and antioxidant profiles of pastures.

#### 4.1.3. Phytochemical Content of the Feeds

GRASS and BLG contained the most chlorophyll A, chlorophyll B, and total phenols while SH contained the least of these compounds. There is a strong positive correlation between chlorophyll A and B and ALA in grasses [59]. Green forages are also known to contain vitamins with antioxidant properties such as vitamin E [56]. The high total phenols levels found in GRASS were expected. It was previously reported that the total phenolic content is higher in grasses than in seeds [60]. In a study comparing a complex pasture mixture to a grain diet, the authors reported higher levels of chlorophyll A, chlorophyll B, and total phenols in pasture [38]. Surprisingly, levels of these antioxidant compounds were not lower in BLG. Drying and fermenting usually decrease concentrations of antioxidants and phenolics [19,31,32]. Tripathi et al. [61] noted that haymaking may cause more leaf dropping and shattering compared to BLG making (leaves are the most nutritious parts of the plant), which may explain why concentrations of these compounds were reduced in HAY but not in BLG. While SH have been investigated for their antioxidant potential [62], our results indicate that SH have low levels of total phenols when compared to GRASS or BLG. However, it appears that the growth stage of the soybean plant affects its phenolic concentration [63].

### 4.2. Animal Performance and Carcass Traits

Results in the present study demonstrate that the diet has an impact on animal growth, carcass traits, and meat quality. Initial body weight did not differ between groups, which may be attributed to pre-trial management. The addition of SH to either GRASS or BLG led to higher final body weight, higher total gain, and higher ADG. While the BLG-SH group was expected to be higher than the other groups for these variables, it was interesting to see a similar trend in the G-SH group which was out on pasture. The BLG-SH group was treated as feedlot cattle. Previous studies showed that cattle finished in feedlots have higher final body weight, total weight gain, and ADG compared to pasture-finished cattle [39,64]. Besides diet, another explanation may be that cattle in feedlots also have less exercise than cattle out on pasture, which reduces their maintenance requirements. The G-SH group was also out on pasture and consumed mostly GRASS, but differences with the BLG-SH group were not significant. Neel et al. [64] showed that increasing the amount of soybean meal and SH in the cattle’s diet led to higher final body weight and ADG. Dennis et al. [65] reported that animals consuming a diet of only HAY showed higher final body weight and ADG than animals consuming a BLG diet. In the present study, no significant differences were seen between the G-HAY and the G-BLG groups. This might be due to animals consuming HAY and BLG as supplemental feeds while eating mostly GRASS. The plant species used might also differ compared to other studies.

Regarding carcass traits, a similar trend was observed for hot carcass weight with the G-SH and the BLG-SH groups weighing more than the other two groups. This finding aligns with previous results about weight gain. Maciel et al. [39] reported that animals in feedlots finished on grain have greater backfat, ribeye size, USDA yield grade, and marbling scores than animals finished on pasture. In the present study, the BLG-SH group showed the same trends, and the same was observed for the G-SH group (which can be attributed to the inclusion of SH). It was previously shown that increasing the amount of soybean meal and SH in the diet increased fat thickness and the USDA yield grade [64]. Supplementing grass-finished cattle with BLG seems to reduce backfat, USDA yield grade, and marbling score. There is limited evidence in the literature demonstrating how feeding conserved forages affects carcass traits and the nutritional profile of beef [19]. The present study indicates that including SH in the diet increases weight gain, while HAY and BLG may reduce weight gain, yield grade, and marbling scores.

### 4.3. Beef Fatty Acids

#### 4.3.1. Saturated Fatty Acid Content of Beef

No differences in SFAs between groups were seen. Red meat and especially beef are criticized for their high SFA content [66]. SFAs increase low-density lipoprotein (LDL) cholesterol, which may increase risks of coronary heart diseases [67]. Based on this, dietary guidelines in the U.S. recommend limiting the intake of SFAs to 10% of daily caloric intake [68]. However, not all SFAs have the same health effects. For instance, palmitic acid (C16:0) has a strong LDL cholesterol-raising effect, while stearic acid (C18:0) has a neutral effect on LDL-cholesterol [69,70]. While no differences were observed in this study, Baublits et al. [37] found that supplementing with SH increases C16:0 levels in beef. Based on the feed FA profile, it was expected that HAY and BLG supplementation would increase the SFA content of beef, especially C16:0. The processing of forages into HAY and BLG generally result in a loss of PUFAs accompanied with an increase in palmitic acid [33,34]. Nevertheless, the lack of significant differences found in the present study might be due to cattle consuming mostly fresh pasture. One limitation of this study is that we did not record the intake of supplemental feeds. Even if the FA profile of the feeds can give us an idea of what to expect in the meat, the gross transfer of dietary SFAs into ruminant products is variable. For example, the transfer of dietary C16:0 into milk fat ranges from 12% to 50% [71].

#### 4.3.2. Monounsaturated Fatty Acid Content of Beef

The MUFA content of beef did not differ between groups. MUFAs make up almost half of beef fat (mostly as oleic acid) [72]. Oleic acid consumption has the potential to lower LDL-cholesterol and blood pressure in humans [69]. Grain-finished beef usually contains up to 70% more MUFAs than GFB [8,73]. In the present study, oleic acid (C18:1 9*c*) was the most abundant FA. The only difference observed was in concentrations of C16:1 9*t* which were lower in beef from the BLG-SH group compared to the other three groups. O’Callaghan et al. [57] observed that adding SH to a concentrate diet resulted in lower MUFA content in milk. Further, Baublits et al. [37] found that supplementing cattle diet with SH led to lower levels of C16:1 9*t*. Ruminant *trans*-FAs are produced by the isomerization of MUFAs in the rumen [74], and grass feeding generally leads to a more favorable rumen pH which allows for more efficient biohydrogenation and isomerization [19,75,76]. Thus, the higher amount of C16:1 9*t* found in the groups with fresh GRASS was expected. The health effects of ruminant *trans*-FAs remain unclear. Some studies reported the antiatherogenic and anticarcinogenic effects of ruminant *trans*-FAs [33], while others reported potential negative health effects [77,78].

#### 4.3.3. Polyunsaturated Fatty Acid Content of Beef

More differences between groups were observed for PUFAs. Overall, beef from the G-HAY group contained more PUFAs than the other three groups. The concentration of *n*-6 PUFAs did not differ between groups, so the variations in PUFA content were due to differences in *n*-3 PUFAs. Both *n*-6 and *n*-3 PUFAs are of interest for human health. Consumption of long-chain *n*-3 PUFAs have anti-inflammatory potential, while *n*-6 PUFAs are generally considered pro-inflammatory [17]. This makes the *n*-6:*n*-3 ratio a crucial metric to determine the health effects of a food [16,17]. In the present study, beef from BLG-SH contained less ALA than beef from G-HAY. This was expected since fresh forages contain 50–75% *n*-3 PUFAs, mostly as ALA [79]. It appears that the addition of SH to the cattle diet reduced the amount of ALA in beef. This finding is supported by results published by Baublits et al. [37]. They reported that the addition of SH to forages resulted in a decrease in *n*-3 PUFAs, especially ALA. Regarding long-chain *n*-3 PUFAs (EPA, DPA, DHA), beef from the G-HAY group contained higher levels of these beneficial FAs than the three other groups. The European Commission considers a food “a source of *n*-3 PUFAs” if 100 g of the food contains at least 40 mg of EPA+DHA or 0.3 g of ALA [52]. Even if the EPA+DHA content in beef of all four groups were below the limit to qualify as a “source of *n*-3 PUFAs”, beef from G-HAY was the closest to meet these standards and can contribute to the intake of these long-chain *n*-3 PUFAs, especially for individuals who have limited access to marine foods [80]. EPA and DHA are linked to healthier cardiovascular, immune, and cognitive functions [81,82]. DPA has been shown to improve cognitive functions, lower cholesterol, and reduce inflammation [83]. Our results indicate that consuming GFB supplemented with HAY provides higher levels of these long-chain *n*-3 PUFAs compared to the other groups. While lower levels of *n*-3 PUFAs in the groups fed SH were expected, it was surprising to see lower levels of these FAs in the groups fed BLG. Haymaking generally results in the loss of PUFAs because of oxidation and the dropping of leaves compared to BLG [19,61]. One explanation might be that animals in the G-BLG group consumed more of their supplemental feed than animals in the G-HAY group. Cattle seem to prefer BLG over HAY [84]. If animals consumed more GRASS in the G-HAY group than the G-BLG group, it might explain the differences in long-chain *n*-3 PUFAs.

The Western diet is generally high in *n*-6 PUFAs and low in *n*-3 PUFAs, leading to increased risks of diseases [16]. The *n*-6:*n*-3 ratio in the Western diet is estimated to be between 15:1 and 20:1. The optimal *n*-6:*n*-3 ratio to benefit human health is between 1:1 and 4:1 [16,17]. In the present study, the *n*-6:*n*-3 ratio was higher in beef from G-SH and BLG-SH, and lower in beef from G-HAY. Even though we noted significant differences between groups, the *n*-6:*n*-3 ratio was still below 2:1 for all of them. The higher *n*-6:*n*-3 ratio in the groups containing SH was expected due to lower amounts of *n*-3 PUFAs and higher levels of *n*-6 PUFAs in SH compared to the other feeds. Duckett et al. [20] found no differences in the *n*-6:*n*-3 ratio when feeding SH to cattle before forage finishing. Baublits et al. [37], on the other hand, reported a greater *n*-6:*n*-3 ratio when cattle where supplemented with SH. However, the addition of any of the supplemental feeds tested cannot explain the wide variations in GFB found by Bronkema et al. [21]. Increasing the *n*-3 PUFA and CLA content while decreasing the SFA and *n*-6 content are priorities to improve the nutritional quality of beef [85].

#### 4.3.4. Biohydrogenation Intermediates in Beef

Biohydrogenation intermediates including CLA, CLnA, and ADs are formed when LA and ALA undergo biohydrogenation in the rumen (70-95% and 85-100%, respectively) [86]. No differences in CLnA and AD were observed in this study. The only difference was seen in levels of C18:2 9*c*,11*t*/9*c*,7*t*, with beef from G-HAY containing the most and beef from BLG-SH containing the least. This was expected since GRASS contains more PUFAs than SH. Feeding mostly GRASS to cattle results in a more favorable rumen pH, leading to more efficient biohydrogenation [75,76]. However, SH has the potential to increase the CLA content of beef compared to grain-diets, mainly because of their high fiber content resulting in a more optimal rumen pH for biohydrogenation to occur [87].

### 4.4. Vitamin E, TBARS, and WBSF

Beef from the three groups fed fresh GRASS contained more vitamin E than beef from BLG-SH. Vitamin E is of interest for human health due to its antioxidant properties [14]. Duckett et al. [20] found no difference in vitamin E levels when pasture was supplemented with SH or not. However, GFB generally contains up to three times more vitamin E than grain-finished beef [15,20,22,51]. The amount of vitamin E found in GFB is enough to protect the beef from oxidation and extend the shelf-life of meat [14,88]. TBARS is an effective assay to measure lipid oxidation. In the present study, TBARS values were higher in beef from BLG-SH compared to the other three groups. Untrained panelists usually do not detect oxidation flavors until oxidation values reach 2.0 mg MDA/kg of tissue [89]. In the current study, only beef from the BLG-SH group exceeded this threshold. Feeding GRASS to cattle usually leads to reduced TBARS values in beef compared to concentrate diets [90]. The higher amounts of antioxidants (including vitamin E) present in GFB might explain the better oxidative stability and lower TBARS values [91].

Shear force values were the lowest for beef from BLG-SH compared to beef from G-HAY and G-BLG. Maciel et al. [39] reported that GFB has higher WBSF values compared to grain-finished beef. These findings indicate that grass-finishing affects the tenderness of beef. Marbling may be a contributing factor to increased meat tenderness (lower WBSF values). Baublits et al. [37] found no difference in shear force values when including SH to a grass diet. Based on the results of the present study, supplementing the diet of cattle with SH might help with meat tenderness, especially in GFB.

## 5. Conclusions

Based on our findings, we can conclude that SH caused more weight gain in cattle, increased the marbling score of beef, and improved the tenderness of GFB. SH did increase the *n*-6:*n*-3 ratio in beef, but it remained under 2:1. The use of SH as a supplemental feed increased TBARS values as well. Feeding GFB fresh GRASS and HAY resulted in a higher PUFA content, especially higher levels of long-chain *n*-3 PUFAs including EPA, DPA, and DHA. Vitamin E concentrations were also increased in beef on fresh pasture, likely contributing to lower TBARS values. In conclusion, none of the supplemental feeds tested in the current study increased the *n*-6:*n*-3 ratio to the values observed previously by Bronkema et al. [21] in their nutritional survey of commercially available GFB. Future research should investigate other feeds (with different plant species) and determine what ingredients cause large increases in the *n*-6:*n*-3 ratio of GFB. As observed here, the *n*-6:*n*-3 ratio of GFB should remain under 4:1 to benefit human health. GFB has the potential to provide beneficial bioactive compounds for human health including long-chain *n*-3 PUFAs, phenols, and vitamin E.

## Figures and Tables

**Table 1:** Mean proximate composition of the feeds.

	GRASS	HAY	BLG ^1^	SH ^2^	*p*-Value
DM ^3^	57.52 ± 22.47	85.82 ± 25.01	82.64 ± 25.02	89.98 ± 25.01	0.159
Ash *	9.20 ± 0.46 ^a^	7.14 ± 0.61 ^b^	8.38 ± 0.63 ^a,b^	4.74 ± 0.61 ^c^	<0.001
CP ^4^ *	15.65 ± 0.39 ^a^	7.15 ± 1.14 ^c^	13.48 ± 1.19 ^a,b^	9.47 ± 1.19 ^b,c^	<0.001
NDF ^5^ *	54.91 ± 3.30 ^b^	66.19 ± 4.01 ^a,b^	54.23 ± 4.04 ^b^	68.29 ± 4.01 ^a^	0.004
ADF ^6^ *	30.84 ± 0.72 ^c^	37.98 ± 1.35 ^b^	33.51 ± 1.39 ^b,c^	51.72 ± 1.35 ^a^	<0.001
Energy ^7^	4566.41 ± 50.99 ^a^	4405.00 ± 90.76 ^a^	4465.41 ± 50.99 ^a^	3709.96 ± 90.76 ^b^	<0.001

Values reported as means ± standard error. Different letters denote statistical significance at *p* < 0.05 (mixed model analysis, post hoc comparison performed using Tukey’s adjustment). *n* = 126 for GRASS, and *n* = 8 for the other three feeds. ^1^ BLG: baleage; ^2^ SH: soybean hulls; ^3^ DM: dry matter (%), * reported in %DM; ^4^ CP: crude protein; ^5^ NDF: neutral detergent fiber; ^6^ ADF: acid detergent fiber; ^7^ Energy (cal/g).

**Table 2:** Mean fatty acid composition of the diets (% total fatty acids).

	GRASS	HAY	BLG ^1^	SH ^2^	*p*-Value
C10:0	0.16 ± 0.26	1.16 ± 0.38	0.14 ± 0.38	0.03 ± 0.38	0.116
C12:0	0.50 ± 0.22 ^b^	1.16 ± 0.23 ^a^	0.49 ± 0.23 ^b^	0.10 ± 0.23 ^c^	<0.001
C13:0	0.01 ± 0.02	0.07 ± 0.03	0.02 ± 0.03	0.00 ± 0.03	0.193
C14:0	0.53 ± 0.29 ^b^	2.14 ± 0.45 ^a^	0.62 ± 0.45 ^a,b^	0.21 ± 0.45 ^b^	0.041
C15:0	0.11 ± 0.06 ^b^	0.49 ± 0.09 ^a^	0.33 ± 0.09 ^a,b^	0.14 ± 0.09 ^b^	0.010
C16:0	14.29 ± 1.98 ^c^	32.16 ± 2.37 ^a^	23.77 ± 2.38 ^b^	14.95 ± 2.37 ^c^	<0.001
C16:1 9*c*	0.23 ± 0.02 ^b^	0.51 ± 0.04 ^a^	0.31 ± 0.04 ^b^	0.23 ± 0.04 ^b^	0.001
C16:1 7*c*	1.19 ± 0.27 ^a^	1.21 ± 0.29 ^a^	1.52 ± 0.29 ^a^	0.11 ± 0.29 ^b^	<0.001
C17:0	0.21 ± 0.02 ^c^	0.57 ± 0.03 ^a^	0.36 ± 0.03 ^b^	0.28 ± 0.03 ^b,c^	<0.001
C18:0	1.57 ± 0.13 ^c^	3.80 ± 0.20 ^a^	2.78 ± 0.20 ^b^	4.33 ± 0.20 ^a^	<0.001
C18:1 9*c*	1.84 ± 0.07 ^c^	3.45 ± 0.23 ^b^	2.43 ± 0.24 ^b,c^	12.61 ± 0.23 ^a^	<0.001
C18:1 11*c*	0.61 ± 0.03 ^c^	1.04 ± 0.09 ^b^	0.72 ± 0.10 ^b,c^	2.43 ± 0.09 ^a^	<0.001
C18:2 *n*-6 (LA) ^3^	12.22 ± 0.35 ^c^	14.86 ± 0.88 ^b,c^	16.42 ± 0.91 ^b^	48.45 ± 0.88 ^a^	<0.001
C18:3 *n*-3 (ALA) ^4^	64.66 ± 1.74 ^a^	32.48 ± 2.57 ^c^	46.47 ± 2.64 ^b^	15.00 ± 2.57 ^d^	<0.001
C20:0	0.58 ± 0.08 ^b^	2.72 ± 0.16 ^a^	1.05 ± 0.16 ^b^	0.41 ± 0.16 ^b^	<0.001
C20:3 *n*-3	0.08 ± 0.03	0.03 ± 0.03	0.05 ± 0.03	0.01 ± 0.03	0.052
C22:0	0.59 ± 0.16 ^b,c^	1.90 ± 0.20 ^a^	1.04 ± 0.21 ^b^	0.29 ± 0.20 ^c^	<0.001
C24:0	0.61 ± 0.32 ^b^	1.78 ± 0.36 ^a^	1.40 ± 0.36 ^a^	0.24 ± 0.36 ^b^	0.001
∑ SFA ^5^	19.17 ± 2.41 ^c^	48.03 ± 3.28 ^a^	31.86 ± 3.29 ^b^	21.01 ± 3.28 ^b,c^	<0.001
∑ OCFA ^6^	0.33 ± 0.06 ^c^	1.11 ± 0.10 ^a^	0.71 ± 0.10 ^a,b^	0.44 ± 0.10 ^b,c^	<0.001
∑ MUFA ^7^	3.87 ± 0.30 ^c^	6.18 ± 0.39 ^b^	4.96 ± 0.40 ^b^	15.40 ± 0.39 ^a^	<0.001
∑ PUFA ^8^	76.96 ± 2.72 ^a^	45.79 ± 3.56 ^c^	63.11 ± 3.58 ^b^	63.51 ± 3.56 ^b^	<0.001
∑ *n*-6 ^9^	12.22 ± 0.35 ^c^	14.86 ± 0.88 ^b,c^	16.42 ± 0.91 ^b^	48.45 ± 0.88 ^a^	<0.001
∑ *n*-3 ^10^	64.73 ± 1.71 ^a^	32.51 ± 2.55 ^c^	46.52 ± 2.62 ^b^	15.02 ± 2.55 ^d^	<0.001
*n*-6:*n*-3 ratio ^11^	0.19 ± 0.04 ^c^	0.53 ± 0.07 ^b^	0.38 ± 0.07 ^b,c^	3.20 ± 0.07 ^a^	<0.001

Values reported as means ± standard error. Different letters denote statistical significance at *p* < 0.05 (mixed model analysis, post hoc comparison performed using Tukey’s adjustment). *n* = 126 for GRASS, and *n* = 8 for the other three feeds. ^1^ BLG: baleage; ^2^ SH: soybean hulls; ^3^ LA: linoleic acid; ^4^ ALA: α-linolenic acid; ^5^ ∑ SFA: total saturated FAs; ^6^ ∑ OCFA: total odd chain FAs; ^7^ ∑ MUFA: total monounsaturated FAs; ^8^ ∑ PUFA: total polyunsaturated FAs; ^9^ ∑ *n*-6: LA; ^10^ ∑ *n*-3: ALA + C20:3 *n*-3; ^11^ *n*-6:*n*-3 ratio: ∑ *n*-6/∑ *n*-3.

**Table 3:** Mean animal performance and carcass traits by diet.

	G-HAY ^1^	G-BLG ^2^	G-SH ^3^	BLG-SH ^4^	*p*-Value
**Growth (kg)**					
Initial BW ^5^	388.70 ± 30.30	390.77 ± 30.33	388.77 ± 30.36	378.27 ± 30.30	0.560
Final BW	483.27 ± 8.63 ^c^	493.74 ± 8.77 ^b,c^	524.33 ± 8.92 ^a,b^	536.31 ± 8.63 ^a^	<0.001
Total gain	94.57 ± 25.64 ^b^	103.02 ± 25.65 ^b^	135.77 ± 25.66 ^a^	158.04 ± 25.64 ^a^	<0.001
ADG ^6^	0.61 ± 0.10 ^b^	0.66 ± 0.10 ^b^	0.88 ± 0.10 ^a^	1.03 ± 0.10 ^a^	<0.001
**Carcass**					
HCW ^7^ (kg)	281.85 ± 5.53 ^c^	287.05 ± 5.62 ^b,c^	311.11 ± 5.72 ^a^	306.53 ± 5.53 ^a,b^	0.003
Backfat (mm)	7.15 ± 0.75 ^a,b^	5.91 ± 0.76 ^b^	9.18 ± 0.77 ^a^	9.38 ± 0.75 ^a^	0.011
Ribeye area (cm^2^)	68.28 ± 2.19 ^b^	75.99 ± 2.20 ^a^	75.57 ± 2.22 ^a^	76.41 ± 2.19 ^a^	0.012
USDA yield grade	2.80 ± 0.30 ^a,b^	2.10 ± 0.31 ^b^	3.29 ± 0.31 ^a^	2.80 ± 0.30 ^a,b^	0.032
Marbling score ^8^	348.00 ± 11.66 ^a,b^	332.55 ± 11.81 ^b^	387.39 ± 11.97 ^a^	392.00 ± 11.66 ^a^	0.004

Values reported as means ± standard error. Different letters denote statistical significance at *p* < 0.05 (mixed model analysis, post hoc comparison performed using Tukey’s adjustment, *n* = 117). ^1^ G-HAY: grass and hay diet; ^2^ G-BLG: grass and baleage diet; ^3^ G-SH: grass and soybean hulls diet; ^4^ BLG-SH: baleage and soybean hulls diet; ^5^ BW: body weight; ^6^ ADG: average daily gain; ^7^ HCW: hot carcass weight; ^8^ Marbling score: 300-Slight-00 and 400-Small-00.

**Table 4:** Mean concentrations of saturated and monounsaturated fatty acids by diet (mg per 100 g beef).

	G-HAY ^1^	G-BLG ^2^	G-SH ^3^	BLG-SH ^4^	*p*-Value
∑ SFA ^5^	275.08 ± 40.89	272.87 ± 41.46	332.71 ± 42.05	356.22 ± 40.89	0.400
C10:0	1.75 ± 1.92	2.54 ± 1.92	3.17 ± 1.92	3.28 ± 1.92	0.163
C12:0	0.57 ± 0.31	0.60 ± 0.31	0.73 ± 0.31	0.70 ± 0.31	0.442
C13:0	0.09 ± 0.07	0.11 ± 0.07	0.12 ± 0.07	0.11 ± 0.07	0.247
C14:0	12.40 ± 2.29	12.04 ± 2.33	14.95 ± 2.36	15.98 ± 2.29	0.566
C15:0	2.00 ± 0.31	2.11 ± 0.31	1.96 ± 0.32	2.02 ± 0.31	0.988
C16:0	162.66 ± 24.15	162.29 ± 24.49	202.57 ± 24.83	220.59 ± 24.15	0.259
C17:0	4.65 ± 0.79	4.86 ± 0.81	5.52 ± 0.82	6.01 ± 0.79	0.617
C18:0	87.03 ± 14.33	84.13 ± 14.49	99.28 ± 14.66	103.06 ± 14.33	0.694
C19:0	1.91 ± 1.54	2.81 ± 1.55	2.84 ± 1.55	2.99 ± 1.54	0.270
C20:0	0.78 ± 0.35	0.67 ± 0.35	0.81 ± 0.35	0.81 ± 0.35	0.305
C22:0	1.23 ± 0.39 ^a^	0.73 ± 0.39 ^b^	0.79 ± 0.39 ^b^	0.84 ± 0.39 ^b^	<0.001
∑ BCFA ^6^	11.11 ± 1.63	11.41 ± 1.65	13.08 ± 1.68	12.29 ± 1.63	0.831
C14:0 *iso*	0.15 ± 0.06	0.19 ± 0.06	0.17 ± 0.06	0.16 ± 0.06	0.863
C15:0 *iso*	0.73 ± 0.16	0.77 ± 0.16	0.94 ± 0.16	0.84 ± 0.16	0.771
C15:0 *anteiso*	0.69 ± 0.15	0.72 ± 0.15	0.73 ± 0.15	0.67 ± 0.15	0.979
C16:0 *iso*	0.77 ± 0.25	0.77 ± 0.25	0.82 ± 0.25	0.80 ± 0.25	0.989
C17:0 *iso*	4.11 ± 0.45	4.16 ± 0.46	4.81 ± 0.47	4.21 ± 0.45	0.689
C17:0 *anteiso*	4.06 ± 0.68	4.27 ± 0.69	4.96 ± 0.70	4.96 ± 0.68	0.717
C18:0 *iso*	0.60 ± 0.19	0.54 ± 0.20	0.65 ± 0.20	0.65 ± 0.19	0.819
∑ MUFA ^7^	313.55 ± 39.66	313.17 ± 40.22	381.51 ± 40.81	371.90 ± 39.66	0.489
∑ *c*MUFA ^8^	276.91 ± 36.21	272.67 ± 36.72	342.84 ± 37.26	342.10 ± 36.21	0.358
C14:1 9*c*	2.67 ± 0.52	2.78 ± 0.53	3.36 ± 0.53	3.13 ± 0.52	0.724
C16:1 9*c*	36.73 ± 4.92	35.81 ± 5.00	45.02 ± 5.09	44.15 ± 4.92	0.444
C16:1 10*c*	4.17 ± 1.55	5.19 ± 1.55	4.51 ± 1.55	4.16 ± 1.55	0.554
C16:1 11*c*	2.00 ± 1.39	2.52 ± 1.39	2.66 ± 1.39	2.76 ± 1.39	0.328
C17:1 9*c*	3.76 ± 0.43	3.98 ± 0.43	4.35 ± 0.44	4.50 ± 0.43	0.490
C18:1 9*c*	199.97 ± 34.69	196.83 ± 35.01	252.79 ± 35.35	255.23 ± 34.69	0.309
C18:1 11*c*	11.87 ± 1.50	10.09 ± 1.51	12.75 ± 1.52	11.57 ± 1.50	0.321
C18:1 12*c*	2.14 ± 0.61	2.12 ± 0.61	2.44 ± 0.61	2.46 ± 0.61	0.560
C18:1 13*c*	2.33 ± 1.60	3.11 ± 1.61	3.64 ± 1.61	3.72 ± 1.60	0.065
C18:1 14*c*	0.91 ± 0.26	0.83 ± 0.26	0.90 ± 0.26	0.90 ± 0.26	0.878
C18:1 15*c*	1.29 ± 0.83	1.74 ± 0.83	1.81 ± 0.83	1.95 ± 0.83	0.153
C20:1 9*c*	2.82 ± 1.70	2.67 ± 1.71	3.08 ± 1.71	2.92 ± 1.70	0.718
C20:1 11*c*	6.26 ± 1.44	4.88 ± 1.44	5.51 ± 1.44	4.63 ± 1.44	0.179
∑ *t*MUFA ^9^	36.64 ± 9.96	40.71 ± 9.99	38.88 ± 10.02	29.80 ± 9.96	0.479
C16:1 9*t*	6.06 ± 1.66 ^a^	6.68 ± 1.66 ^a^	6.11 ± 1.67 ^a^	3.82 ± 1.66 ^b^	0.003
C16:1 10,11,12*t*	5.49 ± 2.51	6.99 ± 2.52	6.83 ± 2.52	6.63 ± 2.51	0.373
C18:1 6-8*t*	1.56 ± 0.99	2.07 ± 0.99	1.83 ± 0.99	2.08 ± 0.99	0.181
C18:1 9*t*	1.62 ± 1.19	2.44 ± 1.19	2.51 ± 1.19	2.52 ± 1.19	0.221
C18:1 10*t*	1.35 ± 1.23	2.49 ± 1.23	2.17 ± 1.23	1.83 ± 1.23	0.149
C18:1 11*t*	13.77 ± 2.73	12.37 ± 2.77	11.73 ± 2.80	5.64 ± 2.73	0.196
C18:1 12*t*	1.36 ± 0.51	1.43 ± 0.51	1.53 ± 0.51	1.26 ± 0.51	0.755
C18:1 13,14*t*	2.92 ± 0.31	2.72 ± 0.31	2.73 ± 0.32	2.51 ± 0.31	0.832
C18:1 15*t*	1.44 ± 1.65	2.24 ± 1.65	2.60 ± 1.65	2.34 ± 1.65	0.079
C18:1 16*t*	1.36 ± 0.35	1.29 ± 0.35	1.27 ± 0.35	1.17 ± 0.35	0.918
∑ FA ^10^	729.92 ± 83.69	698.46 ± 84.92	833.36 ± 86.21	840.46 ± 83.69	0.550

Values reported as means ± standard error. Different letters denote statistical significance at *p* < 0.05 (mixed model analysis, post hoc comparison performed using Tukey’s adjustment, *n* = 117). ^1^ G-HAY: grass and hay diet; ^2^ G-BLG: grass and baleage diet; ^3^ G-SH: grass and soybean hulls diet; ^4^ BLG-SH: baleage and soybean hulls diet; ^5^ ∑ SFA: total saturated FAs; ^6^ ∑ BCFA: total branched chain FAs; ^7^ ∑ MUFA: total monounsaturated FAs; ^8^ ∑ *c*MUFA: total *cis*-monounsaturated FAs; ^9^ ∑ *t*MUFA: total *trans*-monounsaturated FAs; ^10^ ∑ FA: all FAs.

**Table 5:** Mean concentration of polyunsaturated fatty acids by diet (mg per 100 g beef).

	G-HAY ^1^	G-BLG ^2^	G-SH ^3^	BLG-SH ^4^	*p*-Value
∑ PUFA ^5^	98.31 ± 3.50 ^a^	72.54 ± 3.56 ^b^	76.57 ± 3.63 ^b^	73.69 ± 3.50 ^b^	<0.001
∑ *n*-3 ^6^	47.29 ± 2.97 ^a^	29.04 ± 2.99 ^b^	26.57 ± 3.01 ^b^	27.20 ± 2.97 ^b^	<0.001
C18:3 *n*-3 (ALA) ^7^	10.63 ± 1.26 ^a^	10.39 ± 1.26 ^a,b^	8.62 ± 1.27 ^a,b^	8.31 ± 1.26 ^b^	0.014
C20:3 *n*-3	0.89 ± 0.41	0.68 ± 0.41	0.74 ± 0.41	0.70 ± 0.41	0.235
C20:5 *n*-3 (EPA) ^8^	9.26 ± 0.38 ^a^	5.68 ± 0.39 ^b^	5.04 ± 0.39 ^b^	5.00 ± 0.38 ^b^	<0.001
C22:5 *n*-3 (DPA) ^9^	24.18 ± 2.82 ^a^	11.09 ± 2.82 ^b^	10.88 ± 2.83 ^b^	11.59 ± 2.82 ^b^	<0.001
C22:6 *n*-3 (DHA) ^10^	2.33 ± 0.54 ^a^	1.43 ± 0.54 ^b^	1.51 ± 0.54 ^b^	1.60 ± 0.54 ^b^	<0.001
∑ *n*-6 ^11^	47.64 ± 2.84	41.67 ± 2.86	48.26 ± 2.88	44.67 ± 2.84	0.108
C18:2 *n*-6 (LA) ^12^	28.23 ± 3.73	25.42 ± 3.73	30.14 ± 3.74	27.37 ± 3.73	0.121
C18:3 *n*-6	0.72 ± 0.46	0.69 ± 0.46	0.76 ± 0.46	0.76 ± 0.46	0.733
C20:2 *n*-6	1.01 ± 0.32	0.76 ± 0.32	0.84 ± 0.32	0.90 ± 0.32	0.087
C20:3 *n*-6	2.20 ± 0.31	2.21 ± 0.31	2.65 ± 0.31	2.53 ± 0.31	0.123
C20:4 *n*-6	10.43 ± 1.19	10.02 ± 1.20	10.63 ± 1.20	10.02 ± 1.19	0.903
C22:4 *n*-6	5.05 ± 1.36 ^a^	2.68 ± 1.36 ^b^	3.35 ± 1.36 ^b^	3.29 ± 1.36 ^b^	<0.001
*n*-6:*n*-3 ratio ^13^	1.03 ± 0.23 ^c^	1.49 ± 0.23 ^b^	1.89 ± 0.23 ^a^	1.70 ± 0.23 ^a,b^	<0.001
C20:3 *n*-9	3.38 ± 0.93 ^a^	1.87 ± 0.93 ^b^	1.79 ± 0.93 ^b^	1.83 ± 0.93 ^b^	<0.001
∑ CLnA ^14^	1.59 ± 1.15	1.56 ± 1.15	1.76 ± 1.15	1.63 ± 1.15	0.690
C18:3 9*c*,11*t*,15*t*	0.80 ± 0.57	0.82 ± 0.57	0.91 ± 0.57	0.84 ± 0.57	0.676
C18:3 9*c*,11*t*,15*c*	0.79 ± 0.58	0.74 ± 0.58	0.85 ± 0.58	0.79 ± 0.58	0.648
∑ AD ^15^	18.30 ± 6.84	17.25 ± 6.84	17.56 ± 6.85	15.84 ± 6.84	0.833
C18:2 11*t*,15*t*	4.26 ± 0.84	3.27 ± 0.84	3.22 ± 0.85	2.65 ± 0.84	0.207
C18:2 9*t*,12*t*	1.82 ± 1.09	2.27 ± 1.09	2.37 ± 1.09	2.21 ± 1.09	0.377
C18:2 9*c*,14*t*/9*c*,13*t*	2.60 ± 1.27	2.69 ± 1.27	2.78 ± 1.27	2.56 ± 1.27	0.944
C18:2 11*t*,15*c*	4.20 ± 0.82	3.48 ± 0.82	3.04 ± 0.83	2.52 ± 0.82	0.189
C18:2 9*c*,16*t*	1.90 ± 0.88	1.87 ± 0.88	2.07 ± 0.88	2.00 ± 0.88	0.715
C18:2 9*c*,15*c*	2.10 ± 1.29	2.35 ± 1.29	2.59 ± 1.29	2.39 ± 1.29	0.587
C18:2 12*c*,15*c*	1.42 ± 0.80	1.31 ± 0.80	1.48 ± 0.80	1.51 ± 0.80	0.589
∑ CLA ^16^	10.45 ± 3.43	8.35 ± 3.44	9.03 ± 3.44	7.11 ± 3.43	0.107
C18:2 9*c*,11*t*/9*c*,7*t*	6.26 ± 1.00 ^a^	4.41 ± 1.00 ^a,b^	4.66 ± 1.00 ^a,b^	3.05 ± 1.00 ^b^	0.015
C18:2 11*t*,13*c*	1.72 ± 0.89	1.51 ± 0.89	1.64 ± 0.89	1.49 ± 0.89	0.570
C18:2 11*t*,13*t*	1.27 ± 0.83	1.28 ± 0.83	1.44 ± 0.83	1.36 ± 0.83	0.580
C18:2 *t*,*t*	1.20 ± 0.80	1.15 ± 0.80	1.27 ± 0.80	1.21 ± 0.80	0.799

Values reported as means ± standard error. Different letters denote statistical significance at *p* < 0.05 (mixed model analysis, post hoc comparison performed using Tukey’s adjustment, *n* = 117). ^1^ G-HAY: grass and hay diet; ^2^ G-BLG: grass and baleage diet; ^3^ G-SH: grass and soybean hulls diet; ^4^ BLG-SH: baleage and soybean hulls diet; ^5^ ∑ PUFA: total polyunsaturated FAs; ^6^ ∑ *n*-3: total omega-3 FAs; ^7^ ALA: α-linolenic acid; ^8^ EPA: eicosapentaenoic acid; ^9^ DPA: docosapentaenoic acid; ^10^ DHA: docosahexaenoic acid; ^11^ ∑ *n*-6: total omega-6 FAs; ^12^ LA: linoleic acid; ^13^ *n*-6:*n*-3 ratio: ∑ *n*-6/∑ *n*-3; ^14^ ∑ CLnA: total conjugated linolenic acid isomers; ^15^ ∑ Atypical Dienes: total non-conjugated linoleic acid isomers; ^16^ ∑ CLA: total conjugated linoleic acid isomers.

**Table 6:** Mean concentrations of vitamin E and minerals by diet (µg per g of beef).

	G-HAY ^1^	G-BLG ^2^	G-SH ^3^	BLG-SH ^4^	*p*-Value
Vitamin E	29.93 ± 1.44 ^a^	28.86 ± 1.46 ^a^	25.62 ± 1.47 ^a^	13.83 ± 1.44 ^b^	<0.001
Selenium	0.44 ± 0.04	0.42 ± 0.04	0.44 ± 0.04	0.45 ± 0.04	0.561
Iron	59.87 ± 7.61	59.65 ± 7.61	60.41 ± 7.62	56.94 ± 7.61	0.422
Copper	1.98 ± 0.07	2.09 ± 0.07	2.07 ± 0.07	1.93 ± 0.07	0.117
Zinc	126.31 ± 3.35	123.35 ± 3.40	123.80 ± 3.45	119.36 ± 3.35	0.545
Manganese	0.92 ± 0.02 ^a^	0.90 ± 0.02 ^a,b^	0.85 ± 0.02 ^b,c^	0.84 ± 0.02 ^c^	0.002

Values reported as means ± standard error. Different letters denote statistical significance at *p* < 0.05 (mixed model analysis, post hoc comparison performed using Tukey’s adjustment, *n* = 117). ^1^ G-HAY: grass and hay diet; ^2^ G-BLG: grass and baleage diet; ^3^ G-SH: grass and soybean hulls diet; ^4^ BLG-SH: baleage and soybean hulls diet.

## Data Availability

The original contributions presented in the study are included in the article/Supplementary Material. Further inquiries can be directed to the corresponding author.

## Supplementary Materials

The following supporting information can be downloaded at: https://www.mdpi.com/article/10.3390/foods11233856/s1, Table S1: Mean concentrations of saturated and monounsaturated fatty acids by diet (% total fatty acids); Table S2: Mean concentrations of polyunsaturated fatty acids by diet (% total fatty acids).
