# Supplementary material for: Effects of Hay, Baleage, and Soybean Hulls Waste Used as Supplemental Feeds on the Nutritional Profile of Grass-Finished Beef
Source: Foods. 2022 Nov 29;11(23):3856. doi: 10.3390/foods11233856 (PMC9741108; doi:10.3390/foods11233856)
Supplement: Supplementary file 1 [file foods-11-03856-s001.zip › Table S1.pdf]

Table S1. Mean concentrations of saturated and monounsaturated fatty acids by diet (% total fatty acids)

	G-HAY [1]	G-BLG [2]	G-SH [3]	BLG-SH [4]	*p*-value
∑ SFA [5]	35.94 ± 1.63 [b]	37.97 ± 1.63 [a,b]	38.21 ± 1.64 [a,b]	41.10 ± 1.63 [a]	0.003
C10:0	0.26 ± 0.22	0.35 ± 0.22	0.34 ± 0.22	0.38 ± 0.22	0.111
C12:0	0.08 ± 0.04	0.08 ± 0.04	0.08 ± 0.04	0.08 ± 0.04	0.994
C13:0	0.01 ± 0.01	0.02 ± 0.01	0.01 ± 0.01	0.01 ± 0.01	0.113
C14:0	1.46 ± 0.11	1.55 ± 0.11	1.58 ± 0.11	1.69 ± 0.11	0.489
C15:0	0.25 ± 0.01 [a,b]	0.28 ± 0.01 [a]	0.22 ± 0.01 [b]	0.22 ± 0.01 [b]	0.020
C16:0	21.36 ± 1.03 [b]	22.60 ± 1.04 [b]	23.36 ± 1.04 [a,b]	25.46 ± 1.03 [a]	< 0.001
C17:0	0.58 ± 0.03 [b]	0.68 ± 0.03 [a]	0.61 ± 0.03 [a,b]	0.69 ± 0.03 [a]	0.034
C18:0	11.34 ± 1.16	11.73 ± 1.17	11.47 ± 1.17	11.95 ± 1.16	0.522
C19:0	0.28 ± 0.23 [b]	0.44 ± 0.23 [a]	0.32 ± 0.24 [a,b]	0.40 ± 0.23 [a,b]	0.018
C20:0	0.12 ± 0.05	0.10 ± 0.05	0.10 ± 0.05	0.10 ± 0.05	0.148
C22:0	0.20 ± 0.06 [a]	0.11 ± 0.06 [b]	0.11 ± 0.06 [b]	0.12 ± 0.06 [b]	0.003
∑ BCFA [6]	1.47 ± 0.17	1.59 ± 0.17	1.48 ± 0.17	1.39 ± 0.17	0.333
C14:0 *iso*	0.02 ± 0.01	0.02 ± 0.01	0.02 ± 0.01	0.02 ± 0.01	0.093
C15:0 *iso*	0.09 ± 0.02	0.10 ± 0.02	0.10 ± 0.02	0.10 ± 0.02	0.656
C15:0 *anteiso*	0.10 ± 0.02	0.10 ± 0.02	0.08 ± 0.02	0.07 ± 0.02	0.088
C16:0 *iso*	0.10 ± 0.04	0.11 ± 0.04	0.09 ± 0.04	0.10 ± 0.04	0.182
C17:0 *iso*	0.57 ± 0.03	0.60 ± 0.03	0.59 ± 0.03	0.50 ± 0.03	0.069
C17:0 *anteiso*	0.52 ± 0.04	0.58 ± 0.04	0.54 ± 0.04	0.55 ± 0.04	0.541
C18:0 *iso*	0.08 ± 0.03	0.07 ± 0.03	0.07 ± 0.03	0.07 ± 0.03	0.336
∑ MUFA [7]	41.96 ± 0.69 [b]	44.27 ± 0.70 [a,b]	45.06 ± 0.70 [a]	43.57 ± 0.69 [a,b]	0.021
∑ *c*MUFA [8]	37.04 ± 2.00 [b]	38.23 ± 2.00 [a,b]	40.45 ± 2.01 [a]	39.77 ± 2.00 [a,b]	0.018
C14:1 9*c*	0.35 ± 0.06	0.38 ± 0.06	0.37 ± 0.06	0.33 ± 0.06	0.484
C16:1 9*c*	4.88 ± 0.29	4.85 ± 0.30	5.32 ± 0.30	4.90 ± 0.29	0.488
C16:1 10*c*	0.62 ± 0.21 [a,b]	0.75 ± 0.21 [a]	0.55 ± 0.21 [b]	0.53 ± 0.21 [b]	0.005
C16:1 11*c*	0.29 ± 0.21	0.39 ± 0.21	0.32 ± 0.21	0.35 ± 0.21	0.115
C17:1 9*c*	0.53 ± 0.06	0.59 ± 0.06	0.54 ± 0.06	0.56 ± 0.06	0.509
C18:1 9*c*	26.31 ± 3.36 [b]	27.43 ± 3.37 [a,b]	29.64 ± 3.37 [a]	29.56 ± 3.36 [a]	0.007
C18:1 11*c*	1.73 ± 0.16 [a]	1.46 ± 0.16 [b]	1.60 ± 0.16 [a,b]	1.14 ± 0.16 [b]	0.001
C18:1 12*c*	0.32 ± 0.08	0.33 ± 0.08	0.31 ± 0.08	0.32 ± 0.08	0.743
C18:1 13*c*	0.35 ± 0.23 [b]	0.48 ± 0.23 [a]	0.43 ± 0.23 [a,b]	0.46 ± 0.23 [a]	0.021
C18:1 14*c*	0.14 ± 0.04	0.13 ± 0.04	0.11 ± 0.04	0.12 ± 0.04	0.087

C18:1 15*c*	0.20 ± 0.12	0.29 ± 0.12	0.24 ± 0.12	0.26 ± 0.12	0.111
C20:1 9*c*	0.43 ± 0.26	0.43 ± 0.26	0.39 ± 0.26	0.39 ± 0.26	0.511
C20:1 11*c*	0.89 ± 0.22 [a]	0.72 ± 0.22 [b]	0.65 ± 0.22 [b,c]	0.57 ± 0.22 [c]	< 0.001
∑ *t*MUFA [9]	4.92 ± 1.51 [a,b]	6.04 ± 1.51 [a]	4.60 ± 1.51 [b]	3.80 ± 1.51 [b]	< 0.001
C16:1 9*t*	0.93 ± 0.23 [a]	1.04 ± 0.23 [a]	0.85 ± 0.23 [a]	0.52 ± 0.23 [b]	< 0.001
C16:1 10,11,12*t*	0.80 ± 0.37 [b]	1.09 ± 0.37 [a]	0.82 ± 0.37 [b]	0.82 ± 0.37 [b]	0.013
C18:1 6-8*t*	0.25 ± 0.15 [a,b]	0.34 ± 0.15 [a]	0.23 ± 0.15 [b]	0.29 ± 0.15 [a,b]	0.037
C18:1 9*t*	0.25 ± 0.17 [b]	0.38 ± 0.17 [a]	0.30 ± 0.17 [a,b]	0.32 ± 0.17 [a,b]	0.026
C18:1 10*t*	0.21 ± 0.17 [b]	0.37 ± 0.17 [a]	0.25 ± 0.17 [a,b]	0.24 ± 0.17 [b]	0.005
C18:1 11*t*	1.48 ± 0.14 [a]	1.60 ± 0.14 [a]	1.19 ± 0.14 [a,b]	0.65 ± 0.14 [b]	< 0.001
C18:1 12*t*	0.19 ± 0.07 [a]	0.21 ± 0.07 [a]	0.17 ± 0.07 [a,b]	0.15 ± 0.07 [b]	0.007
C18:1 13,14*t*	0.41 ± 0.03 [a]	0.41 ± 0.03 [a]	0.34 ± 0.03 [a,b]	0.31 ± 0.03 [b]	0.002
C18:1 15*t*	0.21 ± 0.27	0.42 ± 0.27	0.30 ± 0.27	0.36 ± 0.27	0.126
C18:1 16*t*	0.18 ± 0.05 [a]	0.19 ± 0.05 [a]	0.15 ± 0.05 [b]	0.14 ± 0.05 [b]	0.030
∑ FA [10]	729.92 ± 83.69	698.46 ± 84.92	833.36 ± 86.21	840.46 ± 83.69	0.550

Values reported as means ± standard error. Different letters denote statistical significance at $p < 0.05$ (mixed model analysis, post-hoc comparison performed using Tukey's adjustment, $n = 117$).

[1] G-HAY: grass and hay diet; [2] G-BLG: grass and baleage diet; [3] G-SH: grass and soybean hulls diet

[4] BLG-SH: baleage and soybean hulls diet

[5] ∑ SFA: all saturated FAs (C10:0-C22:0, even and odd)

[6] ∑ BCFA: sum of all branched chain FAs (*iso*14:0, *iso*15:0, *anteiso*15:0, *iso*16:0, *iso*17:0, *anteiso*17:0, *iso*18:0)

[7] ∑ MUFA: all monounsaturated FAs (14:1, 16:1, 17:1, 18:1, 20:1)

[8] ∑ *c*MUFA: 14:1, 17:1, sum of *c*16:1, *c*18:1, and *c*20:1

[9] ∑ *t*MUFA: sum of *t*16:1 and *t*18:1

[10] ∑ FA: sum all of FAs in mg/100 g meat
