# Supplementary material for: Effects of Hay, Baleage, and Soybean Hulls Waste Used as Supplemental Feeds on the Nutritional Profile of Grass-Finished Beef
Source: Foods. 2022 Nov 29;11(23):3856. doi: 10.3390/foods11233856 (PMC9741108; doi:10.3390/foods11233856)
Supplement: Supplementary file 1 [file foods-11-03856-s001.zip › Table S2.pdf]

Table S2. Mean concentrations of polyunsaturated fatty acids by diet (% total fatty acids)

	GHAY [1]	GBLG [2]	GSH [3]	BLGSH [4]	*p*-value
∑ PUFA [5]	16.04 ± 1.20 [a]	11.79 ± 1.21 [a,b]	11.56 ± 1.22 [a,b]	10.44 ± 1.20 [b]	0.020
∑ *n*-3 [6]	7.68 ± 0.56 [a]	4.61 ± 0.56 [b]	3.92 ± 0.56 [b]	3.78 ± 0.56 [b]	< 0.001
C18:3 n-3 (ALA) [7]	1.64 ± 0.22 [a]	1.64 ± 0.22 [a]	1.29 ± 0.22 [a,b]	1.14 ± 0.22 [b]	0.015
C20:3 *n*-3	0.14 ± 0.06 [a]	0.11 ± 0.06 [a,b]	0.09 ± 0.06 [b]	0.10 ± 0.06 [b]	0.005
C20:5 *n*-3 (EPA) [8]	1.55 ± 0.09 [a]	0.91 ± 0.10 [b]	0.77 ± 0.10 [b]	0.71 ± 0.09 [b]	< 0.001
C22:5 *n*-3 (DPA) [9]	3.96 ± 0.49 [a]	1.76 ± 0.49 [b]	1.57 ± 0.49 [b]	1.61 ± 0.49 [b]	< 0.001
C22:6 *n*-3 (DHA) [10]	0.39 ± 0.07 [a]	0.20 ± 0.07 [b]	0.20 ± 0.07 [b]	0.22 ± 0.07 [b]	< 0.001
∑ *n*-6 [11]	7.80 ± 0.89	6.88 ± 0.90	7.41 ± 0.90	6.39 ± 0.89	0.573
C18:2 *n*-6 (LA) [12]	4.57 ± 0.82	4.18 ± 0.82	4.63 ± 0.82	3.91 ± 0.82	0.668
C18:3 *n*-6	0.12 ± 0.07	0.12 ± 0.07	0.10 ± 0.07	0.11 ± 0.07	0.416
C20:2 *n*-6	0.16 ± 0.03 [a]	0.10 ± 0.03 [b]	0.11 ± 0.03 [b]	0.10 ± 0.03 [b]	0.034
C20:3 *n*-6	0.37 ± 0.04	0.36 ± 0.04	0.39 ± 0.04	0.36 ± 0.04	0.927
C20:4 *n*-6	1.76 ± 0.30	1.68 ± 0.30	1.73 ± 0.30	1.46 ± 0.30	0.725
C22:4 *n*-6	0.83 ± 0.21 [a]	0.43 ± 0.21 [b]	0.46 ± 0.21 [b]	0.46 ± 0.21 [b]	< 0.001
n-6:*n*-3 ratio [13]	1.03 ± 0.23 [c]	1.49 ± 0.23 [b]	1.89 ± 0.23 [a]	1.70 ± 0.23 [a,b]	< 0.001
C20:3 *n*-9	0.56 ± 0.14 [a]	0.31 ± 0.14 [b]	0.23 ± 0.14 [b]	0.26 ± 0.14 [b]	0.001
∑ CLnA [14]	0.26 ± 0.17	0.26 ± 0.17	0.22 ± 0.17	0.23 ± 0.17	0.422
C18:3 9*c*,11*t*,15*t*	0.13 ± 0.09	0.13 ± 0.09	0.12 ± 0.09	0.12 ± 0.09	0.502
C18:3 9*c*,11*t*,15*c*	0.13 ± 0.09	0.12 ± 0.09	0.11 ± 0.09	0.11 ± 0.09	0.287
∑ AD [15]	2.61 ± 1.03 [a]	2.64 ± 1.03 [a]	2.14 ± 1.03 [a,b]	2.11 ± 1.03 [b]	0.008
C18:2 11*t*,15*t*	0.55 ± 0.13 [a]	0.47 ± 0.13 [a,b]	0.37 ± 0.13 [b,c]	0.34 ± 0.13 [c]	< 0.001
C18:2 9*t*,12*t*	0.28 ± 0.16	0.36 ± 0.16	0.30 ± 0.16	0.30 ± 0.16	0.074
C18:2 9*c*,14*t*/9*c*,13*t*	0.39 ± 0.19	0.43 ± 0.19	0.34 ± 0.19	0.34 ± 0.19	0.156
C18:2 11*t*,15*c*	0.54 ± 0.13 [a]	0.49 ± 0.13 [a]	0.35 ± 0.13 [b]	0.32 ± 0.13 [b]	< 0.001
C18:2 9*c*,16*t*	0.30 ± 0.13	0.30 ± 0.13	0.27 ± 0.13	0.27 ± 0.13	0.489
C18:2 9*c*,15*c*	0.32 ± 0.18	0.37 ± 0.18	0.31 ± 0.18	0.32 ± 0.18	0.237
C18:2 12*c*,15*c*	0.23 ± 0.12	0.22 ± 0.12	0.20 ± 0.12	0.21 ± 0.12	0.662
∑ CLA [16]	1.50 ± 0.52 [a]	1.25 ± 0.52 [b]	1.10 ± 0.52 [b,c]	0.93 ± 0.52 1	< 0.001
C18:2 9*c*,11*t*/9*c*,7*t*	0.83 ± 0.14 [a]	0.61 ± 0.14 [b]	0.52 ± 0.15 [b]	0.37 ± 0.14 [c]	< 0.001
C18:2 11*t*,13*c*	0.27 ± 0.13 [a]	0.24 ± 0.13 [a,b]	0.21 ± 0.13 [a,b]	0.21 ± 0.13 [b]	0.029
C18:2 11*t*,13*t*	0.21 ± 0.12	0.21 ± 0.12	0.19 ± 0.12	0.19 ± 0.12	0.554
C18:2 *t*,*t*	0.19 ± 0.12	0.19 ± 0.12	0.16 ± 0.12	0.17 ± 0.12	0.322

Values reported as means ± standard error. Different letters denote statistical significance at $p < 0.05$ (mixed model analysis, post-hoc comparison performed using Tukey's adjustment, n = 117).

[1]GHAY: grass and hay diet; [2] GBLG: grass and baleage diet; [3] GSH: grass and soybean hulls diet;
[4] BLGSH: baleage and soybean hulls diet

[5] ∑ PUFA = LA + ALA + GLA + Eicosadienoic + Eicosatrienoic + DGLA + Mead + Arachidonic + EPA + DTA + DPA *n*-3 + DHA

[6] ∑ *n*-3 = ALA + EPA + DHA + DPA *n*-3 + Eicosatrienoic

[7] ALA: α-linolenic acid; [8] EPA: eicosapentaenoic acid; [9] DPA: docosapentaenoic acid;

[10] DHA: docosahexaenoic acid;

[11] ∑ *n*-6 = LA + GLA + Eicosadienoic + DGLA + Arachidonic + DTA; [12] LA: linoleic acid;

[13] *n*-6:*n*-3 ratio = ∑ *n*-6/∑ *n*-3

[14] ∑ CLnA = sum of conjugated linolenic acid isomers (*c*9, *t*11, *t*15 18:3 + *c*9, *t*11, *c*15 18:3)

[15] ∑ Atypical Dienes = sum of non-conjugated linoleic acid isomers (*t*11, *t*15 18:2 + *t*9, *t*12 18:2 + *c*9, *t*14/*c*9, *t*13 18:2 + *t*11, *c*15 18:2 + *c*9, *t*16 18:2 + *c*9, *c*15 18:2 + *c*12, *c*15 18:2)

[16] ∑ CLA = sum of conjugated linoleic acid isomers (*c*9, *t*11/*t*7, *c*9 18:2 + *t*11, *c*13 18:2 + *t*11, *t*13 18:2 + *t,t* 18:2)
